# Percutaneous Application of Galvanic Current in Rodents Reverses Signs of Myofascial Trigger Points

**DOI:** 10.1155/2020/4173218

**Published:** 2020-05-28

**Authors:** R. Margalef, M. Bosque, P. Monclús, P. Flores, F. Minaya-Muñoz, F. Valera-Garrido, M. M. Santafé

**Affiliations:** ^1^Unit of Histology and Neurobiology, Department of Basic Medical Sciences, Faculty of Medicine and Health Sciences, Rovira i Virgili University, Carrer St. Llorenc¸No. 21, 43201 Reus, Spain; ^2^MVClinic Institute, Madrid, Spain; ^3^CEU San Pablo University, Madrid, Spain; ^4^Getafe C.F., Madrid, Spain

## Abstract

An increase in the spontaneous release of acetylcholine (ACh) at the motor endplate is directly related to the generation of myofascial trigger points (MTrPs). In this study, percutaneous electric fields were applied to an animal model of MTrPs with high levels of spontaneous ACh release. All experiments were performed on Swiss mice and Sprague Dawley rats. For evaluating the spontaneous neurotransmission, intracellular recordings were performed, and the frequency of miniature endplate potentials was evaluated. Electromyographic recordings were also conducted to evaluate the endplate noise. Finally, the number and strength of local twitch responses (LTR) were evaluated using ultrasound recordings. The protocols used for the electric currents were 0.4 mA for five seconds and four repetitions (protocol 1), 1.5 mA for five seconds and three repetitions (protocol 2), and 3 mA for three seconds and three repetitions (protocol 3). After a subcutaneous injection of neostigmine (NTG), a great increase was observed in the frequency of mEPPs, together with an elevated endplate noise. Protocols 2 and 3 were the most effective. Protocol 3 could completely reverse the action of NTG at both three hours and 24 hours, respectively. The application of percutaneous currents produced both an increase in the number (144%) and in the speed (230% faster) of LTR compared with dry needling. In conclusion, higher doses of electrical current are more effective for decreasing MTrPs findings in an animal model.

## 1. Introduction

Myofascial trigger points (MTrPs) can generate a characteristic type of muscle pain called myofascial pain syndrome (MPS), encompassing sensory, motor, and autonomic symptoms [1, 2]. MTrPs are hyperirritable nodules within taut bands of skeletal muscle responsible. MPS is very common in humans, and it is estimated that almost everyone might suffer an episode at least once during their lifetime [3].

An increase in ACh released spontaneously at the level of the motor endplate is directly related to the generation of myofascial trigger points (MTrPs) [1, 4]. The increase in spontaneously released ACh may be recorded electromyographically via a characteristic recording known as endplate noise [5, 6]. We have recently reported the accumulation of glycosaminoglycans (GAGs) in the area of MTrPs [4]. These GAGs can trap substances, such as nociceptive and sensitize substances, contributing, in part, to the clinical symptoms of MPS [7].

One of the therapeutic interventions employed in the treatment of MTrPs is dry needling (DN). This consists of the use of mechanical stimuli applied via a needle to either eliminate or inactivate the MTrP and thus obtain therapeutic benefits [8]. One of the possible benefits is the destruction of the dysfunctional neuromuscular junctions that cause the MTrPs [1] and, consequently, the replacement of these synapses by nerve regeneration [9]. Moreover, this local muscle destruction involves an inflammatory reaction [9] that can remove the GAGs, together with trapped substances. During the DN of the MTrP, a visible contraction of a part of the muscle, known as the local twitch response (LTR), is often elicited [10]. Some authors suggest that DN is more effective if an LTR is obtained during the procedure [10–12]. There are studies that obtain clinical benefits without the existence of LTR. For example, Hakim et al. [13] working with patients with active MTrP in the upper trapezius muscle obtained that the DN without causing LTR has superiority over the DN along with causing LTR in clinical parameters that include pain, pressure threshold, and the range of lateral flexion movement of the neck. If the LTR occurs, the contraction induced in the area of the MTrP could help to clear nociceptive substances associated with GAGs reduction of pain.

The use of percutaneous electric currents is increasing in modern medicine [14, 15]. Electric currents can accelerate muscle regeneration [16] and tendon repair [17], as well as influence the growth of several cancer cells [18]. Moreover, several studies have reported that cells can directionally respond to electric fields, applied in both in vitro and in vivo settings, via a phenomenon called electrotaxis [19]. Endothelial electrotaxis is also involved in angiogenesis [20], and the application of percutaneous electric currents can affect inflammatory mediators in damaged muscle tissue and promote a fresh vascularization of the injured area [21]. Thus, both the angiogenesis and electrotaxis of inflammatory cells may be beneficial for the treatment of MTrPs. Furthermore, electric currents cause muscle contraction, which is secondary to depolarization of muscular fibers and motor axons [22, 23], thus contributing to immediate reduction in pain. Till date, only a few studies have been conducted on the use of percutaneous electrical fields on MTrP.

The main objective of this study is to evaluate if the different forms of electrical currents commonly used in clinical interventions can be useful in the treatment of MPS. For that, a model of myofascial trigger points (MTrPs) is obtained when neostigmine is injected in mice. In this model, the neuromuscular neurotransmission is increased. Using percutaneous electric currents, the action of neostigmine is reversed at both three and 24 hours. Moreover, the application of electric currents produced an increase in the number and in the speed of local twitch responses compared to dry needling.

## 2. Materials and Methods

### 2.1. Animals

Experiments were performed on young adult male Swiss mice (45 to 50 days postnatal, *n* = 35) and Sprague Dawley rats (60 to 70 days postnatal, *n* = 6; Charles River, L'Arbresle, France). All experiments were carried out in the vivarium and in the neurophysiology laboratory of the Faculty of Medicine of the Rovira i Virgili University. Animals were housed in standard MAKROLON^R^ cages (27 × 27 × 14 cm3 for mice and 52 × 28 × 15 cm^3^ for rats), and two animals were distributed for each cage. Temperature was maintained at 20°C–22°C by an electronic thermostat, and a relative humidity was fixed at 60–70%. The circadian rhythms were 12 hours of white neon light and 12 hours of darkness. Feeding and hydration of animals has been *ad libitum*, based on the VRF-1 mouse and A.04 PANLAB^R^ rat feed supplied by (PANLAB, Spain) and chlorinated regular tap water. The animals were cared for in accordance with the guidelines of the European Community's Council Directive of 24th November, 1986 (86/609/EEC) and the Spanish Royal Decree 53/2013 for the humane treatment of laboratory animals. This study was approved by the Ethics Committee of the Rovira i Virgili University.

### 2.2. Muscles

Animals were anaesthetized with 2% tribromoethanol (0.15 ml/10 g body weight, I.P.) and put to death by exsanguination while deeply anaesthetized. Tribromoethanol has been chosen because it is a rapidly reversible anesthetic that does not affect the peripheral nervous system. The levator auris longus (LAL) was excised and dissected on a Sylgard-coated Petri dish containing normal Ringer solution continuously bubbled with 95% O_2_/5% CO_2_. The LAL muscle was used for the electrophysiological study, and the gastrocnemius muscles were used for electromyography and ultrasonographic recordings.

### 2.3. Anticholinesterasic Exposure

Neostigmine methyl sulfate (NTG; 0.1 mg NTG/kg body weight; Sigma) was injected subcutaneously (thoracolumbar area) into the adult male Swiss mice. To ensure the correct drug administration, a cholinergic syndrome should appear within 30 minutes posttreatment (for further details, see Margalef et al. [4]).

### 2.4. Procedure with Electric Currents

**Figure 1:**
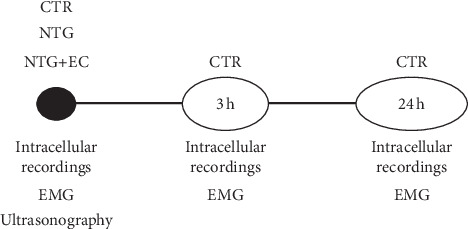
Timeline of the experimental procedure. CTR, control; NTG, neostigmine; EC + NTG, electric currents applied to animals treated with neostigmine. In the lower part of the line there are experimental methodologies: intracellular recordings in mice; EMG, electromyography in mice; Ultrasonography is performed in rats.

Figure 1 shows the timeline and the experimental procedure used. Thirty minutes after injecting the neostigmine, electric/galvanic current was applied directly to the gastrocnemius muscles and LAL. The parameters analyzed were intensity, time in seconds, and the number of repetitions. These were described by three numerical values in the same order (for example, 3 mA for three seconds and three repetitions, i.e., 3:3:3) [24]. The protocols with continuous direct current used were 0.4 mA for five seconds and four repetitions (protocol 0.4:5:4), 1.5 mA for five seconds and three repetitions (protocol 1.5:5:3), and 3 mA for three seconds and three repetitions (protocol 3:3:3). Additionally, some experiments have been carried out using microcurrent stimulation: the intensity for 10 minutes was set at 10^−6 ^mA and the frequency at 10 Hz (10^−6 ^mA/10 Hz/10 min). A specifically developed medically certified device (Physio Invasiva®, PRIM Physio, Spain) was used for application of electrical current. The electromyography record and the intracellular recordings were taken three hours and 24 hours after applying the currents.

### 2.5. Electrophysiology: Intracellular Recordings

Spontaneous miniature endplate potentials (mEPPs) were recorded intracellularly with conventional glass microelectrodes filled with 3M KCl (20–40 MΩ resistance). Records were rejected if the membrane potential (Vm) was < −50 mV or if it fell by more than 5 mV during the recording period.

The recording electrodes were connected to an amplifier (Tecktronics, AMS02). A distant Ag–AgCl electrode connected to the bath solution via an Agar bridge (Agar 3.5% in 137 mMNaCl) was used as a reference. The MEPPs were digitized (DIGIDATA 1200 Interface, Axon Instruments Inc, CA), stored, and analyzed using a computer. The Axoscope 10.2 was used (Axon Instruments Inc.) for data acquisition and analysis. The MEPP frequency was recorded for 100s from at least 15 different neuromuscular junctions, and the mean values were determined. The mean amplitude (mV) per fiber was calculated and corrected for nonlinear summation [25], assuming a membrane potential of −80 mV. We proceeded as follows: NTG was injected subcutaneously into adult male Swiss mice. When the acute cholinergic syndrome disappeared (30 minutes), the sample was normally excised and dissected as explained above, and mEPPs were recorded from at least 15 different neuromuscular junctions.

### 2.6. Endplate Noise Recordings

The needle EMG (nEMG) records were always obtained from an anaesthetized animal at a controlled room temperature of between 22°C and 25.8°C. The muscle used for this study was the gastrocnemius due to its easy access and suitability for the study. Registers were obtained using an electromyography system (MedelecMystro plus, GR20) with a monopolar EMG needle (Natus Manufacturing Limited). The needle was slowly inserted into the muscle, and once inside, it was moved to record in all directions. The muscle was divided into twelve areas to cover the entire muscle and to avoid registering the same endplate noise twice. The recording needle was introduced into the gastrocnemius until an audible change in sound was heard. The electromyography screen was then studied, and if it was correct (without an alternating current, artifacts, etc.), the endplate noise was recorded. The number of areas with endplate noise (maximum 12) was recorded. While the endplate noise was being recorded, we studied the frequency to know the number of potentials per second that appeared, expressed in Hz.

The experiments were performed as follows: (1) subcutaneous injection of NTG, (2) the animal displayed cholinergic syndrome, (3) a control nEMG recording was performed in the left gastrocnemius, and (4) nEMG examination of the right gastrocnemius muscle.

### 2.7. Ultrasonography

Muscular ultrasonography was performed in rats using an ultrasound device (General Electric, LOGIQ E R7) and a transducer (General Electric, L10-22-RS). A LTR was considered in the event of a sudden and localized movement occurring when the needle was inserted into the muscle (see examples by Margalef et al. [4]). Ultrasound needle within tissues is identified as a bright line that penetrates tissue or only as a reflection of the tip. If a sudden and very localized movement when the needle is inserted into the muscle is seen, it is considered to be a local twitch response (LTR). That LTR can be viewed with ultrasound technique as a very discreet area (see supplemental videos). This area is called the “LTR area.” If the needle insertion only shows the tip deforming the tissue while penetrating, it is considered that the local twitch response has not occurred. First, rats were treated with neostigmine, and after 30 minutes, the muscles were palpated in order to identify an area with a taut band. In these areas, LTRs occurred in response to the insertion of a solid filament needle in both legs. Next, three needle insertions were performed, one insertion per second, in the left gastrocnemius. Thereafter, the same procedure was performed in the right gastrocnemius in order to identify a taut band area. When the area was identified, the needle was inserted, and an electric current protocol was applied (protocol 1.5:5:3). Initially, an abrupt contraction of the entire muscle is observed through the application of percutaneous electrical currents. After that, LTRs appear as highly localized movements in a very discrete area of the muscle.

**Figure 2:**
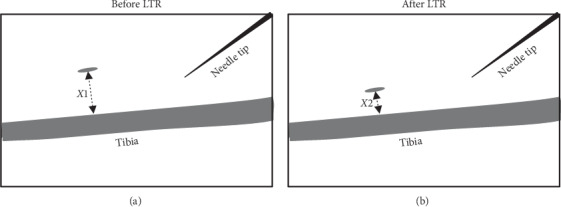
Determination of speed of the local twitch response. The local twitch response (LTR) can be seen by ultrasound technique as a very discreet area (gray ellipse; see supplementary videos). This area is called the “LTR area.” After determining the LTR area, the distance from the point at the beginning of the LTR and the tibia (X1) and the distance from the point at the end of the LTR and the tibia (X2) were measured. In all cases, three measurements were made. X1-X2 = distance traveled by the LTR during the spasm. The total LTR duration was extracted from the video analyzers. These parameters were used to calculate the speed (*v*) of LTR: *v*= (X1−X2)/LTR duration.

During both procedures, ultrasound images were recorded and subsequently analyzed at low speed, using the Kinovea movement analysis program (https://www.kinovea.org/). The intensity of the LTR was evaluated using image analysis. We assessed the rate of change as an indicator of power. This was done as follows (see Figure 2): once an LTR area was identified, we measured the distance from both points, at the beginning of the LTR and the tibia, and the distance from the point the LTR ended and the tibia (average of three measurements). The subtraction of these two values is known as the LTR-distance. The LTR duration extracted from video analyzers was used to calculate the speed of the LTR:

(1) $$\begin{array}{c} v\;=\;\frac{\text{distance before the LTR }-\text{ distance after the LTR}}{\text{LTR duration}}. \end{array}$$

### 2.8. Statistical Procedure

Values are expressed as means ± SEM. Sometimes, the values are expressed as “percentage of change.” This is defined as: (experimental value/control value) *×* 100. We used the two-tailed Welch's *t*-test for unpaired values because our variances were not equal. We prefer this test as it is more conservative than the ordinary *t*-test. Differences were considered significant at *P* < 0.05.

## 3. Results

In this study, an animal model of MTrPs was used, generated by the subcutaneous injection of neostigmine (NTG). In this animal model of MTrPs, several clinical signs of MTrPs can be observed: an elevated endplate noise compared to the norm, as evidenced by the electromyographic recording; many muscular fibers with contraction knots (narrower sarcomeres and locally thickened muscle fiber) surrounded by the infiltration of connective tissue, such as molecules of glycosaminoglycan; and palpable taut bands and LTRs in response to needle stimulation.

### 3.1. Intracellular Recording

To rule out a possible adverse effect of the electrical currents, initially, spontaneous neurotransmission in healthy muscles was evaluated. By applying electrical currents in healthy muscles, the frequency of miniature endplate potentials (mEPPs) was neither modified after three hours (% change: 2.40% ± 5.88, *P* > 0.05, *N* = 5 muscles, 75 fibers) nor 24 hours thereafter (% change: 12.45 % ± 11.32, *P* > 0.05, *N* = 3 muscles, 45 fibers).

**Figure 3:**
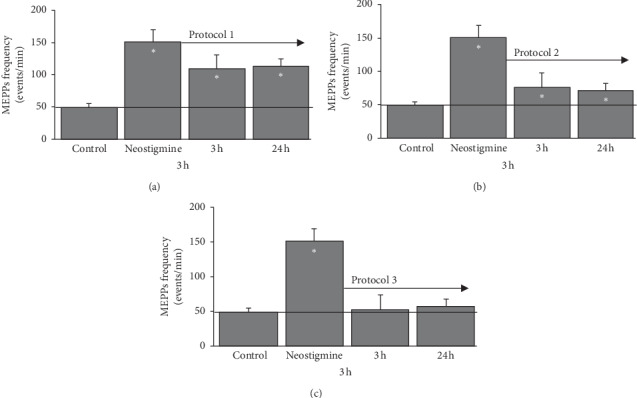
Intracellular recordings. After the subcutaneous injection of neostigmine, several electric current protocols have been applied: (a) protocol 1 (4 repetitions of 0.4 mA for 5 seconds), (b) protocol 2 (3 repetitions of 1.5 mA for 5 seconds), and (c) protocol 3 (3 repetitions of 3 mA for 3 seconds). The recordings were made 3 hours and 24 hours after applying the protocols. The values are expressed in events per minute (mean ± SEM). Control = 7 muscles; 66 fibers were recorded in total. Neostigmine = 5 muscles; 45 fibers recorded in total. (neostigmine + protocol 1) = 3 muscle; 39 fibers were recorded in total. (neostigmine + protocol 2) = 4 muscles; 36 fibers were recorded in total. (neostigmine + protocol 3) = 4 muscles; 71 fibers were recorded in total. (neostigmine + protocol 1) = 3 muscles; 40 fibers recorded in total. (neostigmine + protocol 2) = 3 muscles; 36 fibers were recorded in total. (neostigmine + protocol 3) = 3 muscles; 56 fibers were recorded in total. ^*∗*^*P* < 0.05with respect to control values.

A high increase in the frequency of mEPPs (about 300%) was obtained in the LAL muscle 30 minutes after a subcutaneous injection of the NTG (0.1 mg NTG/kg body weight) (Figure 3). The mEPPs were only reduced to 30% with the 0.4:5:4 protocol and to 50% (Figure 3(a)) with the 1.5:5:3 protocol of electric currents (Figure 3(b)). The 3:3:3 protocol completely reverses the frequency of the mEPPs to control values (Figure 3(c)). When the frequency of mEPPs was evaluated 24 hours later, similar values were obtained. The mEPPs frequency was slightly reduced (≈27%) by the 0.4:5:4 protocol, 1.5:5:3 reduced it slightly more (≈50%), and the complete reversion was achieved with the 3:3:3 protocol (Figure 3).

Lower intensity protocols were also used, such as 10^−6 ^mA 10^ ^Hz-100^ ^*μ*s, for a longer duration (10 minutes), which is a common protocol used in clinical practice. This protocol was revealed to be ineffective, as the frequency of mEPPs was still more elevated (≈235%) at three hours compared to those observed in the controls (mEPPs frequency, experiment: 114.99 ± 23.35; controls: 48.87 ± 5.28; *n* = 3 animals, minimum of 15 fibers per muscle; *P* < 0.05) and at 24 hours (mEPPs frequency, experiment: 124.32 ± 20.43; controls: 48.87 ± 5.28; *n* = 3 animals, minimum of 15 fibers per muscle, *P* < 0.05).

### 3.2. Electromyography

Similar to what was observed in the intracellular recordings, electric currents in healthy muscles did not change the number of areas with endplate noise after either three hours (% variation: 15.21% ± 8.23 *P* > 0.05, *N* = 5 mice) or 24 hours (% variation: 1.43% ± 7.05, *P* > 0.05, *N* = 3 mice). The frequency of endplate noise was not modified in any control group (% variation at 3h: 0.53% ± 5.66, *N* = 5 mice; % variation at 24 hours: 4.04% ± 2.80, *N* = 3 mice; *P* > 0.05 in both cases).

**Figure 4:**
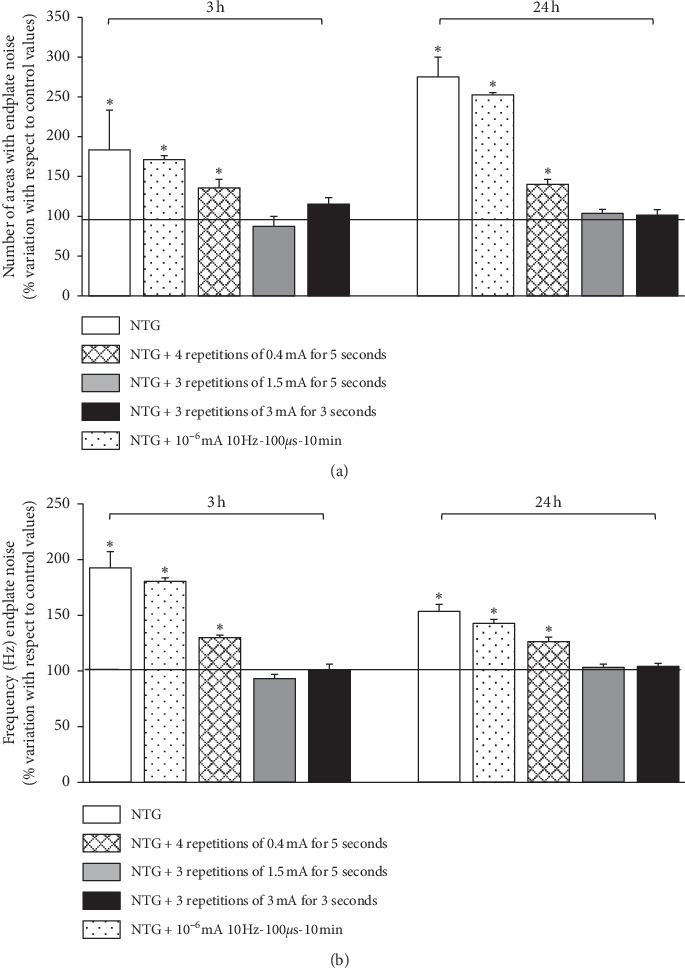
Electromyography. (a) Number of areas with endplate noise. (b) Endplate noise frequency. On the left, 3h after injection of neostigmine (NTG). On the right, 24 h after injection of neostigmine. Values are expressed in % of variation between experimental values registered in the right leg and control values registered in the left leg (mean ± SEM). For each experimental series, *n* = 5 animals (5 control gastrocnemius and 5 gastrocnemius treated). ^*∗*^*P* < 0.05 with respect to control values.

Electromyography recordings revealed an elevated endplate noise in the gastrocnemius muscle of animals treated with neostigmine. The results of electromyography coincide with the results obtained in the intracellular recording experiments using electric currents (Figure 4). Protocols 1.5:5:3 and 3:3:3 completely reversed the action of neostigmine at three hours, and this effect was maintained 24 hours later. This action was identical for both the number of areas with endplate noise and the frequency of events in each of the areas with endplate noise. However, the 0.4:5:3 protocol reduced the number of areas with endplate noise and the frequency, although it did not reverse these completely (Figure 4).

When applying the 10^−6 ^mA, 10 Hz, 100µs during the 10 minutes protocol, the areas with endplate noise and the mean frequency at each of these areas were still elevated at three hours (% of change: 172.17 ± 5.0 and 180.3 ± 3.2, respectively, *n* = 3 animals, *P* < 0.05) and at 24 hours (% of change: 262 ± 2.0 and 146.1 ± 3.5, respectively, *n* = 3 animals, *P* < 0.05).

### 3.3. Ultrasonography

**Figure 5:**
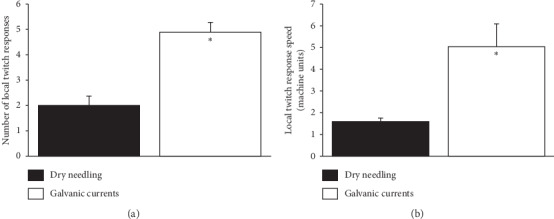
Ultrasonography. (a) Average number of local twitch responses obtained with dry needling and galvanic current. (b) Speed obtained for each local twitch response during the dry needling technique and after applying galvanic current. The values are expressed in machine units (mean ± SEM). *N* = 6 rats. ^*∗*^*P* < 0.05 for values obtained with galvanic current compared to values obtained using the dry needling technique.

Thirty minutes after the NTG injection and after identifying the taut band, the DN was performed with multiple quick insertions using a solid filament needle, from which about two LTRs were removed in the right gastrocnemius (1.59 ± 0.16). Subsequently, another solid filament needle was inserted into the left gastrocnemius, until an LTR was obtained. Then, electrical currents were applied through that needle. After a massive contraction of the entire muscle, about five extremely fast LTRs were observed (5.04 ± 1.05). Percutaneous electrical currents (protocol 1.5:5:3) produced a 144% increase in LTRs compared to dry needling (Figure 5(a)). In addition, the speed of each LTR was 230% faster with the electric currents than with DN (Figure 5(b)).

## 4. Discussion

This study evaluated the effects of percutaneous electric currents on an animal model of MTrPs. Several protocols were employed with varying levels of intensity and duration: 10^−6 ^mA 10 Hz-100 *μ*s during 10 minutes, 0.4 mA for five seconds and four repetitions, 1.5 mA for five seconds and three repetitions, and 3 mA for three seconds of and three repetitions. Only the latter protocol completely reversed the high spontaneous neurotransmission achieved with a subcutaneous neostigmine injection to control values. Thus, the number of MEPPs returned to normal values at three hours and remained normal for 24 hours. In addition, the electromyography records also reversed the high values of both the number of areas with endplate noise and the frequency of endplate noise. The four protocols were effective at different degrees. 1.5 mA for 5 seconds and three repetitions and 3 mA for three seconds and three repetitions were the most effective. In addition, the LTRs elicited by electrical currents compared to those produced by DN have been evaluated and demonstrated to be far more effective electric currents.

Simons' integrated hypothesis [26] postulates that an increase in spontaneous neuromuscular neurotransmission is responsible for a whole cascade of events, resulting in the generation and maintenance of MTrPs. Based on this hypothesis, we have generated MTrPs in mice by increasing the amount of ACh in the synapses with an anticholinesterase agent [4]. In this model, we obtained an increase in the spontaneous release of ACh evidenced by both intracellular electrophysiological recordings of the MEPPs and the electromyography recordings of endplate noise. It is well known that electric currents cause an extensive depolarization of the axonal terminals [27]. In this situation, all the available synaptic vesicles are released, and the nervous terminals can be exhausted for a period lasting from a few minutes to hours [28]. Subsequently, all the components of neurotransmission recover their initial values and the release of ACh returns to normal. Note that this situation is maintained for 24 hours. In terms of lifespan, 24 hours, in rodents, is a period comparable to ∼30–40 days for humans [28]. The electric currents are effective even at situations of long (24 hours) high neurotransmission induced by neostigmine, which may be associated with consolidated structural changes. The different degrees of effectiveness of the protocols used demonstrate that the intensity of the electric current is important in order to achieve clinical results. In this sense, protocol 3 (repetitions of 3 mA for three seconds) is shown to be the most effective in all the situations studied.

Glycosaminoglycans (GAGs) are macromolecules with a high hygroscopic capacity (to absorb and retain liquid and other substances) synthesized by fibroblasts [29]. Accumulations of GAGs in the MTrPs have been observed [4]. These GAGs can trap substances such as protons, SP, CGRP, bradykinin, TNF-*α*, IL-1*β*, IL-6, IL-8, 5-HT, and norepinephrine which have been found in the area of MTrPs [7]. These substances may be responsible for part of the clinical symptoms of MPS [7]. An LTR or a contraction of the whole muscle may compress the GAGs and release these substances to the vascular system and “wash out” the MTrP area. A visible contraction of part of the muscle after mechanical stimulation of an MTrP can occur (LTR) [10]. This mechanical stimulus can be produced either with a needle during DN or after the application of percutaneous electric currents. The LTR should not be confused with the abrupt contraction of the entire muscle by the application of percutaneous electric currents. When the needle is inserted into the muscle, the LTR is a sudden and highly localized movement produced into the muscle. When an electric current is applied, an immediate contraction of the muscle as a whole is obtained. Then, LTRs spontaneously appear. It is suggested that it is reasonable to associate the LTR with clinical improvement. Thus, the first authors to use ultrasound to identify, visualize, and quantify the number of LTRs caused during dry needling treatments in patients [30] reported that the group in which LTRs were elicited experienced a higher reduction of the pain compared to the group that did not show LTR. More recently, Koppenhaver et al. [31] conducted a study evaluating the relationship between LTR and pain, dysfunction, and nociceptive sensation with the functionality in the lower lumbar musculature. These authors concluded that the patients who experienced LTRs showed better improvement than the patients who did not. However, these improvements did not affect pain values. According to Koppenhaver and colleagues, the presence of LTRs during needling may be relevant; however, this is not indicative of successful treatment. If the presence of LTRs is clinically advantageous, a greater number of LTRs, with a greater power, is clinically relevant. However, there is no uniformity of criteria in reference to the clinical significance of the LTR. There are studies showing that there is a decrease in pain without evident LTR after treating with DN (see, for example, Hakim et al. [13]). Moreover, according to Perrault et al. [10], LTR during DN seems unnecessary to control myofascial pain and is not related to many of the positive effects of DN.

The effects of percutaneous electric currents may be further explained by other physiological phenomena, such as electrotaxis or galvanotaxis. These changes can cause a net movement of cells, such as endothelial cells [20], lymphocytes [32], or macrophages [33] towards the cathode. Moreover, endothelial electrotaxis is also involved in angiogenesis [20]. Inflammatory cells such as lymphocytes [32] or macrophages [33] migrate toward the cathode in physiologically relevant electric fields. In the case of the muscle, the application of percutaneous electric currents in damaged muscle tissue can especially affect inflammatory mediators and influence the new vascularization of the injured area [21]. The angiogenesis and the electrotaxis of inflammatory cells may also be beneficial for treating MTrPs.

In summary, percutaneous electric currents, applied with sufficient intensity and power, offer several physiopathological explanations to support MTrPs: (1) the depletion of the synaptic vesicles caused by a massive depolarization of the axonal terminals, with a subsequent normalization of the neurotransmission; (2) LTR and the contraction of the entire muscle which may compress the GAGs and “wash out” the nociceptive substances; (3) electrotaxis or galvanotaxis of inflammatory cells and angiogenesis. These effects, at multiple neurochemical, electrical, and mechanical levels, provide new insights for novel therapeutic purposes.

## 5. Conclusion

A model of myofascial trigger points (MTrPs) is obtained when neostigmine is injected in mice. In a model of myofascial trigger points, the neuromuscular neurotransmission is increased. Using percutaneous electric currents, the action of neostigmine is reversed at both three and 24 hours. Moreover, the application of electric currents produced an increase in the number and in the speed of local twitch responses compared to dry needling. In conclusion, higher doses of electrical current are more effective for decreasing MTrPs findings in an animal model.

## Data Availability

The (Figures 3, 4, and 5) data used to support the findings of this study are included within the article.

## Supplementary Materials

Supplementary Materials Supplemental videos: two videos showing muscular echography and several local twitch responses after treatment. DN: dry needling. GC: galvanic current.
