# Supplementary material for: Percutaneous Application of Galvanic Current in Rodents Reverses Signs of Myofascial Trigger Points
Source: Evid Based Complement Alternat Med. 2020 May 28;2020:4173218. doi: 10.1155/2020/4173218 (PMC7275229; doi:10.1155/2020/4173218)
Supplement: Supplementary Materials — Supplemental videos: two videos showing muscular echography and several local twitch responses after treatment. DN: dry needling. GC: galvanic current. [file 4173218.f1.zip › 4173218.f1/Supplemental videos.docx]

Supplemental videos: Two videos showing muscular echography and several local twitch responses after treatment:

DN: dry needling.

GC: galvanic current.
